# Identification and Validation of Three Hub Genes Involved in Cell Proliferation and Prognosis of Castration-Resistant Prostate Cancer

**DOI:** 10.1155/2022/8761112

**Published:** 2022-08-17

**Authors:** Pan Yu, Yibei Dai, Tingting Zhuang, Xiaofang Yue, Yuhua Chen, Xuchu Wang, Xiuzhi Duan, Ying Ping, Yiyi Xie, Ying Cao, Zhihua Tao

**Affiliations:** Department of Laboratory Medicine, The Second Affiliated Hospital of Zhejiang University School of Medicine, Hangzhou, Zhejiang 310009, China

## Abstract

**Background:**

The acquisition of castration resistance is lethal and inevitable in most prostate cancer patients under hormone therapy. However, effective biomarkers and therapeutic targets for castration-resistant prostate cancer remain to be defined.

**Methods:**

Comprehensive bioinformatics tools were used to screen hub genes in castration-resistant prostate cancer and were verified in androgen-dependent prostate cancer and castration-resistant prostate cancer in TCGA and the SU2C/PCF Dream Team database, respectively. Gene set enrichment analysis and in vitro experiments were performed to determine the potential functions of hub genes involved in castration-resistant prostate cancer progression.

**Results:**

Three hub genes were screened out by bioinformatics analysis: MCM4, CENPI, and KNTC1. These hub genes were upregulated in castration-resistant prostate cancer and showed high diagnostic and prognostic value. Moreover, the expression levels of the hub genes were positively correlated with neuroendocrine prostate cancer scores, which represent the degree of castration-resistant prostate cancer aggression. Meanwhile, in vitro experiments confirmed that hub gene expression was increased in castration-resistant prostate cancer cell lines and that inhibition of hub genes hindered cell cycle transition, resulting in suppression of castration-resistant prostate cancer cell proliferation, which confirmed the gene set enrichment analysis results.

**Conclusions:**

MCM4, CENPI, and KNTC1 could serve as candidate diagnostic and prognostic biomarkers of castration-resistant prostate cancer and may provide potential preventive and therapeutic targets.

## 1. Background

In recent years, prostate cancer has been the second leading cause of cancer-related death in males [1]. Hormone therapy that targets the androgen receptor (AR) remains the mainstay therapy for prostate cancer. Unfortunately, nearly all patients under hormone therapy ultimately progress to castration-resistant prostate cancer (CRPC), which is characterized by its nonresponse to most currently available therapies, and the median overall survival time is only 15-36 months [2, 3]. The treatment of CRPC remains a significant challenge, and there is still a lack of excellent prognostic biomarkers and effective treatments for CRPC patients [2, 4, 5]. Therefore, the identification and validation of hub genes involved in CRPC are necessary to provide new insights into prediction, prognostic evaluation, and therapeutic strategies for CRPC.

Identification of differentially expressed genes (DEGs) among expression profiles is a crucial step in searching for hub genes and is widely used to explore potential mechanisms of cancer progression [6, 7]; gene expression arrays have long been a ubiquitous platform used to identify DEGs [8]. However, differences in sample size, sample sources, or platforms can lead to inconsistency in the expression levels of genes identified among different studies [9–11]. Therefore, eliminating the differences in individual studies and ensuring the reliability and representativeness of the selected DEGs have become a top priority. A bioinformatics method, named robust rank aggregation (RRA), that can integrate multiple gene expression datasets has been developed to settle this problem [12]. This method can be used to analyze a series of separate datasets by aggregating the resultant DEG list in each dataset to minimize bias and errors among different datasets, thereby obtaining robust DEGs representing multiple integrated expression datasets. To date, many studies have exploited the RRA method to obtain robust DEGs for identification of hub genes [11, 13]. However, in prostate cancer, most studies have focused on seeking DEGs between adjacent normal tissues and prostate cancer (PCa) tissues and seldom concentrate on finding robust DEGs between androgen-dependent prostate cancer (ADPC) and CRPC [14, 15].

Most prostate cancer patients undergoing androgen deprivation therapy will inevitably develop CRPC, which involves multiple mechanisms and signaling pathways, and the progression is complicated [16, 17]. The existing biomarkers of CPRC mainly focus on genomic aberrations, and several biomarker-driven clinical trials are underway in patients with CRPC. However, in addition to genomic aberrations, the phenotype of CRPC tumors can evolve during disease progression, and treatment resistance manifests by changes in gene expression, epigenetics, and/or tumor morphology [18]. Therefore, much effort should be made to explore and illustrate the potential mechanisms driving CRPC development and to provide new biomarkers and therapeutic targets for CRPC. Our study imaginatively integrated GEO transcriptome datasets from different studies to screen robust DEGs in CRPC and combined them with a series of bioinformatics tools to identify potential hub genes, enriching the types of biomarkers associated with transcriptome expression changes. Gene set enrichment analysis (GSEA) and in vitro experiments were performed to explore and verify the potential biological functions of hub genes in CRPC. We found that MCM4, CENPI, and KNTC1 were overexpressed in CRPC cells and that increased MCM4, CENPI, and KNTC1 expression predicted poor overall survival in CRPC patients. A decrease in the expression of the three hub genes led to abnormal CRPC cell cycle transition and suppressed CRPC cell proliferation. In addition, the expression levels of the three hub genes were associated with the NEPC score, indicating that the three hub genes may be involved in CRPC progression. Our study demonstrates that the three identified hub genes may be potential therapeutic targets in CRPC. Suppression of these genes may provide a new treatment strategy for CRPC. In addition, combined with other prostate cancer biomarkers, such as prostate specific antigen (PSA), and serum androgen levels, these hub genes could provide a novel approach to predict CRPC.

## 2. Materials and Methods

### 2.1. Collection of PCa Gene Expression Datasets

All available datasets were downloaded from GEO. The selection criteria were as follows: (1) the gene expression data of both ADPC and CRPC tissue samples derived from clinical patients were included, and the gene expression data of xenograft-derived tumor tissue samples were excluded; (2) a minimum of five ADPC and five CRPC tissue samples were included in an independent gene expression dataset; and (3) the GEO platform contained at least 5,000 gene probes. Selected by the above criteria, three datasets were finally included in this study: GSE3325, GSE32269, and GSE35988. Additionally, RNA-sequencing and clinical data of ADPC were obtained from TCGA database (https://portal.gdc.cancer.gov/repository), and RNA-sequencing and clinical data of CRPC were downloaded from SU2C/PCF Dream Team (http://www.cbioportal.org/study/summary?id=prad_su2c_2019) [19]. The TCGA and SU2C/PCF data were normalized, and the batch effects were removed using the R package “limma”. The preprocessing data have been validated in published research [20].

### 2.2. Identification of Robust DEGs

The R package “limma” was utilized to normalize the expression data and find DEGs in each GEO dataset with the cutoff criteria of |log2 − fold change (FC)| ≥ 1 and adjusted *P* value < 0.05 [21]. Then, the RRA method was applied to integrate DEGs from each dataset, which is a standard method to minimize bias and errors among several datasets. The ranking of each gene in the final gene list of was determined by its *P* value, and genes with an adjusted *P* < 0.05 were considered to indicate significant DEGs in RRA analysis [12].

### 2.3. Functional Enrichment Analysis

The Database for Annotation, Visualization and Integrated Discovery (DAVID) (https://david.ncifcrf.gov/) was used to conduct Gene Ontology (GO) function and Kyoto Encyclopedia of Genes and Genomes (KEGG) enrichment analyses of robust DEGs [22]. GO terms and KEGG pathways with adjusted *P* < 0.05 were considered statistically significant and visualized using “ggplot2” in R package [23].

### 2.4. Protein–Protein Interaction Network Construction

The STRING database (https://cn.string-db.org/) was employed to construct a protein–protein interaction (PPI) network [24]. The parameter of interactive relationships among DEGs was set as high confidence > 0.7. Visualization and analysis of the PPI network were performed using Cytoscape software version 3.4.0 (http://www.cytoscape.org/) [25]. The plug-in MCODE of Cytoscape software was used to screen key modules in the whole PPI network (the parameters were set to default), and pathway enrichment analysis was performed for DEGs in each module using the DAVID database. The cytoHubba plug-in provides twelve topological analysis methods for ranking nodes in a network by their network features. However, considering that the MCC algorithms can capture more essential proteins in the top ranked list among both high-degree and low-degree proteins, the MCC algorithms of cytoHubba plug-in were carried out to screen potential hub genes in the whole PPI network in this study [26].

### 2.5. Gene Set Enrichment Analysis

The 75th percentile and 25th percentile values of the expression level of each hub gene were set as the cutoff points for dividing the data into a high expression group and a low expression group for CRPC samples. GSEA_4.0.1 software (http://www.gsea-msigdb.org/gsea/index.jsp) was utilized to conduct GSEA between the two groups to determine the potential functions of the three hub genes in CRPC. The annotated gene set c2.cp.kegg.v7.5.1.symbols.gmt (system files of GSEA software) was selected as the reference gene set. Under the cutoff criterion of a nominal (NOM) *P* value < 0.05, the pathways with the highest normalized enrichment score (NES) ranking were chosen for analysis.

### 2.6. Cells and Cell Culture

Three human castration-resistant prostate adenocarcinoma cell lines [PC-3 (catalog no: TCHu158), DU145 (catalog no: TCHu222), and 22-Rv1(catalog no: TCHu100)] and one human androgen-dependent prostate adenocarcinoma cell line [LNCaP (catalog number: TCHu173)] were purchased from the Cell Bank of Shanghai Institute of Biochemistry and Cell Biology, Chinese Academy of Sciences (Shanghai, China), a distributor of ATCC. All four PCa cell lines were cultured in a 5% CO2 incubator in RPMI 1640 medium with 10% FBS. The ATCC (https://www.atcc.org/) was used to determine the culture method for each cell line.

### 2.7. siRNA Transient Transfection

Cells were seeded at 2 × 10^5^ cells/well in 6-well plates and transfected with siRNA using Lipofectamine 2000 according to the manufacturer's instructions (catalog no: 11668019, Invitrogen Life Technologies, Carlsbad, CA). After four hours of transfection, the medium containing siRNA was replaced with a fresh medium. The target sequences of siRNAs are listed in Table 1.

### 2.8. Western Blotting, Cell Proliferation, and Cell Cycle Distribution Analysis

Western blotting, cell viability assays, and cell cycle distribution analysis were performed according to our previous study [27]. Antibodies against CENPI (1: 2000, catalog no: ab118796) and MCM4 (1 : 2000, catalog no: ab4459) were purchased from Abcam (Cambridge, MA, USA). An antibody against KNTC1 (1 : 200, catalog no: sc-81853) was purchased from Santa Cruz (Santa Cruz, CA, USA). An antibody against GAPDH (1 : 1000, catalog no: 5174S) was purchased from Cell Signaling Technology (Beverly, MA, USA). HRP-conjugated secondary antibodies derived from rabbits and mice were also purchased from Cell Signaling Technology [anti-rabbit IgG (1 : 5000, catalog no: 7074S) and anti-mouse IgG (1 : 5000, catalog no: 7076S)]. Cell viability was measured using a Cell Counting Kit-8 (catalog no: C008-3, 7seabiotech, Shanghai, China) following the manufacturer's instructions. Cell cycle distribution was measured via flow cytometry with PI/RNase staining buffer (catalog no: 550825, BD Biosciences).

### 2.9. Statistical Analysis

The Mann–Whitney test, Kruskal–Wallis test, and Student's *t* test were employed to determine the statistical significance of differences between groups. Receiver-operating characteristic (ROC) curves were plotted, and the area under the ROC curve (AUC) was calculated to assess the diagnostic values of the hub genes. Kaplan–Meier analysis for overall survival was executed based on the hub gene expression levels using GraphPad prism 9 software (https://www.graphpad.com/). The cutoff value was set at the mean value to divide CRPC samples into a high expression group and a low expression group, and a log-rank test was used to determine statistical significance. In addition, the association of hub genes with clinical features (Gleason score, serum PSA and NEPC score) of CRPC were analyzed with Spearman *r* or Pearson *r*. All statistical tests were two-sided and performed with GraphPad Prism 9 software; statistical significance was defined as a *P* value < 0.05.

## 3. Results

### 3.1. Robust Rank Aggregation Analysis of DEGs between ADPC and CRPC

**Figure 1:**
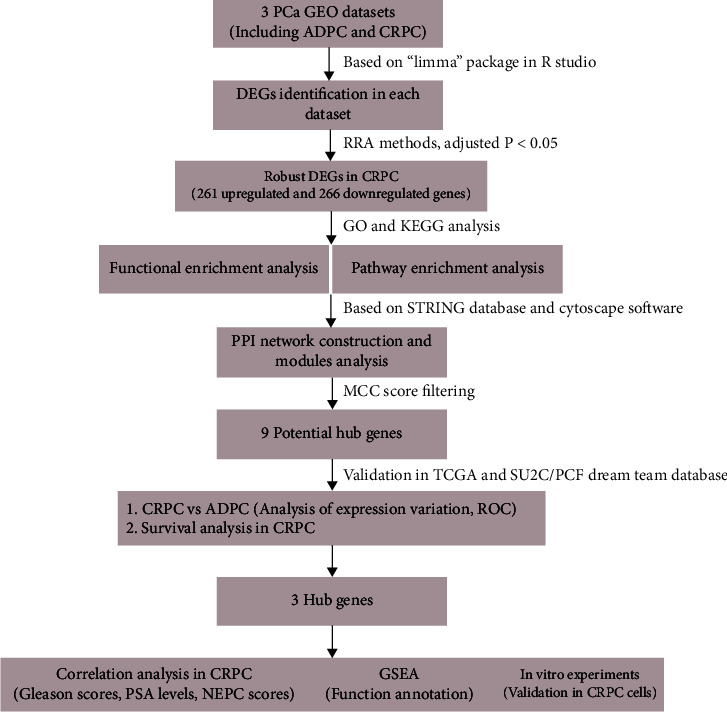
Study workflow. GEO: Gene Expression Omnibus; DEG: differentially expressed genes; RRA: robust rank aggregation; GO: Gene Ontology; KEGG: Kyoto Encyclopedia of Genes and Genomes; PPI: protein-protein interaction network; MCC: maximal clique centrality; TCGA: The Cancer Genome Atlas; SU2C/PCF Dream Team: SU2C-Prostate Cancer Foundation Prostate Dream Team; ROC: receiver-operating characteristic curve; NEPC: neuroendocrine prostate cancer; GSEA: gene set enrichment analysis.

In this study, three available Gene Expression Omnibus (GEO) gene expression profiles were downloaded according to strict criteria (see the “Materials and Methods”), and the characteristics of the GEO datasets are shown in Table 2. Subsequently, identification, validation, and functional analysis of DEGs were performed in line with the workflow (Figure 1).

**Figure 2:**
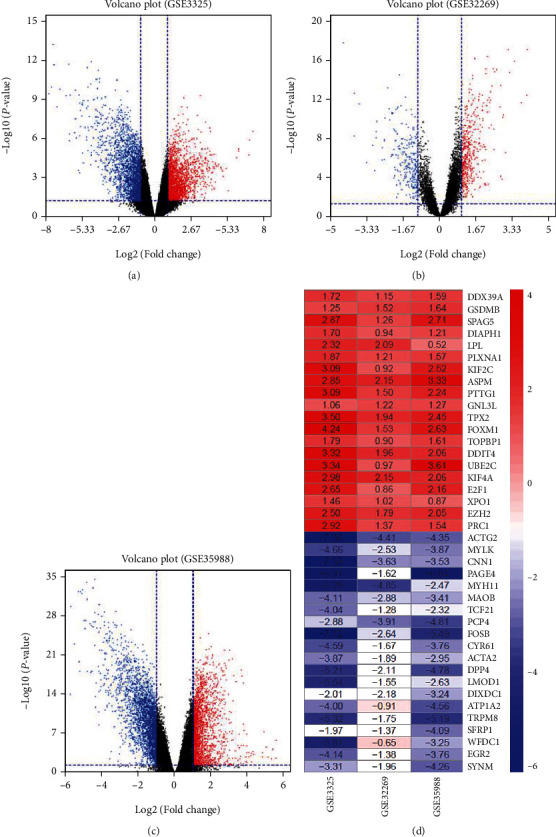
Identification of DEGs in each GEO dataset. (a–c) Volcano plots of the distribution of DEGs in each dataset. (d) Expression heatmap of the top20 significant upregulated and downregulated DEGs determined by *P* value. Each row represents one gene, and each column indicates one dataset. Red means upregulation, and blue means downregulation. The numbers in the heatmap indicate logarithmic fold change in each dataset calculated by the “limma” R package. DEG: differentially expressed gene; GEO: Gene Expression Omnibus; RRA: robust rank aggregation.

First, the R package “limma” was utilized to normalize the data and find DEGs of each GEO dataset between CRPC and ADPC [21]. Volcano plots of the distribution of DEGs in each dataset are shown in Figures 2(a)–2(c). The RRA method was used to integrate DEGs from each dataset and obtain the robust DEGs. A total of 261 upregulated and 266 downregulated genes were screened out, and top 20 significantly upregulated and downregulated robust DEGs are shown in a heatmap in Figure 2(d). DDX39A was the most significantly upregulated gene (*P* = 3.59*E* − 06, average logarithmic fold change = 1.487), followed by GSDMB (*P* = 3.69*E* − 05, average logarithmic fold change = 1.47). In addition, ACTG2 (*P* = 1.40*E* − 07, average logarithmic fold change = 5.37) and MYLK (*P* = 2.34*E* − 07, average logarithmic fold change = 3.69) were the most significantly downregulated genes.

### 3.2. Functional Enrichment Analysis of Robust DEGs

**Figure 3:**
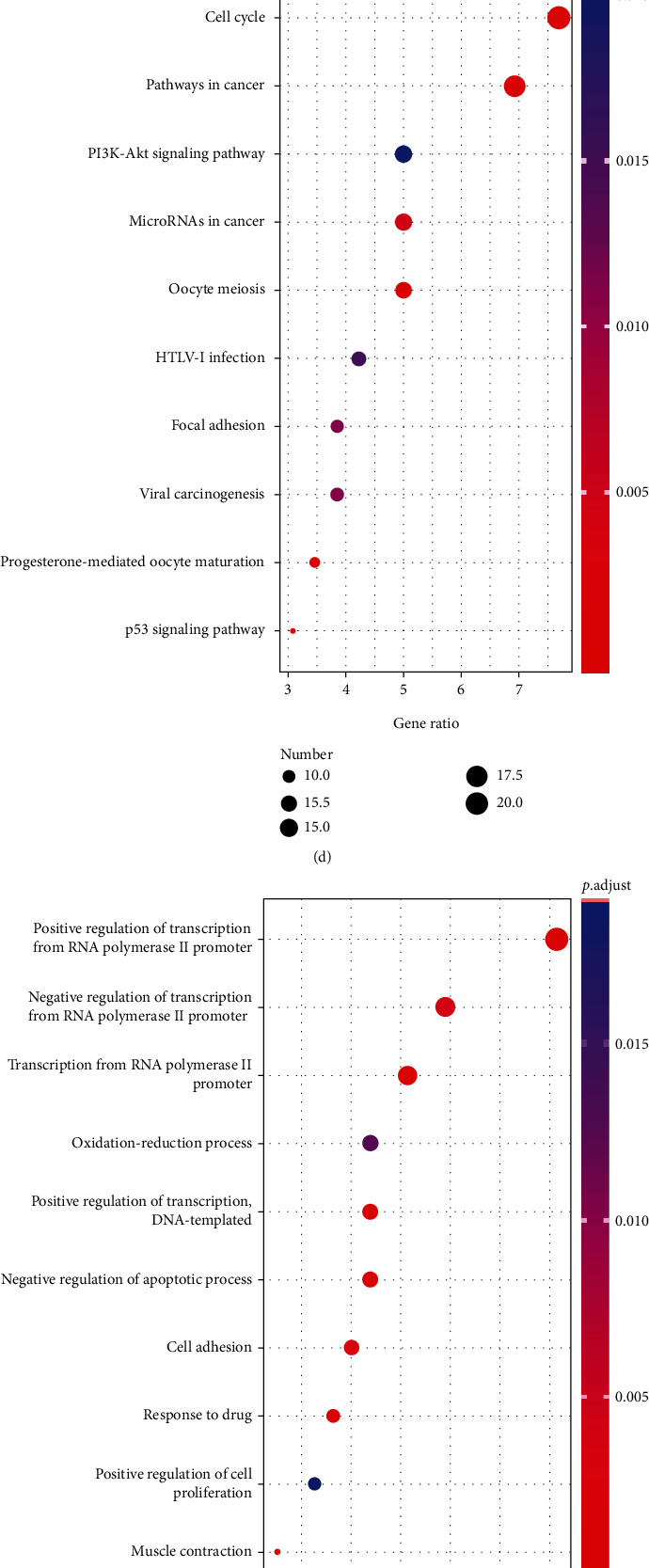
GO and KEGG enrichment analysis of robust DEGs. (a–c) Bubble plot of GO enrichment analysis of upregulated DEGs. (d) Bubble plot of KEGG pathway enrichment analysis of upregulated DEGs. (e–g) Bubble plot of GO enrichment analysis of downregulated DEGs. (h) Bubble plot of KEGG pathway enrichment analysis of downregulated DEGs. GO: Gene Ontology; KEGG: Kyoto Encyclopedia of Genes and Genomes.

To determine the potential functional roles of the upregulated and downregulated robust DEGs, the DAVID database was used to analyze GO function and KEGG enrichment. The top 10 enriched GO terms and KEGG pathways of the above robust DEGs are shown in Figure 3. In biological processes (BP), the upregulated robust DEGs were significantly enriched in cell division, mitotic nuclear division, and cell proliferation (Figure 3(a)); the downregulated robust DEGs were significantly enriched in positive regulation of transcription from the RNA polymerase II promoter, negative regulation of transcription from the RNA polymerase II promoter, and transcription from the RNA polymerase II promoter (Figure 3(e)). For cell component (CC), nucleus, cytoplasm, and nucleoplasm were the most enriched terms in the upregulated robust DEGs (Figure 3(b)), and the downregulated robust DEGs were mostly enriched in the cytoplasm, extracellular exosome, and cytosol (Figure 3(f)). Molecular function (MF) analysis revealed that the upregulated robust DEGs were mainly enriched in protein binding, ATP binding, and DNA binding (Figure 3(c)), and the downregulated robust DEGs were mostly enriched in sequence-specific binding, heparin binding, and actin binding (Figure 3(g)). Moreover, in KEGG analysis, the upregulated robust DEGs were significantly enriched in the cell cycle, pathways in cancer, and the PI3K-Akt signaling pathway (Figure 3(d)), and the downregulated robust DEGs were significantly enriched in pathways in cancer, HTLV-I infection, and focal adhesions (Figure 3(h)).

### 3.3. Construction of a PPI Network of Robust DEGs and Identification of Potential Hub Genes

**Figure 4:**
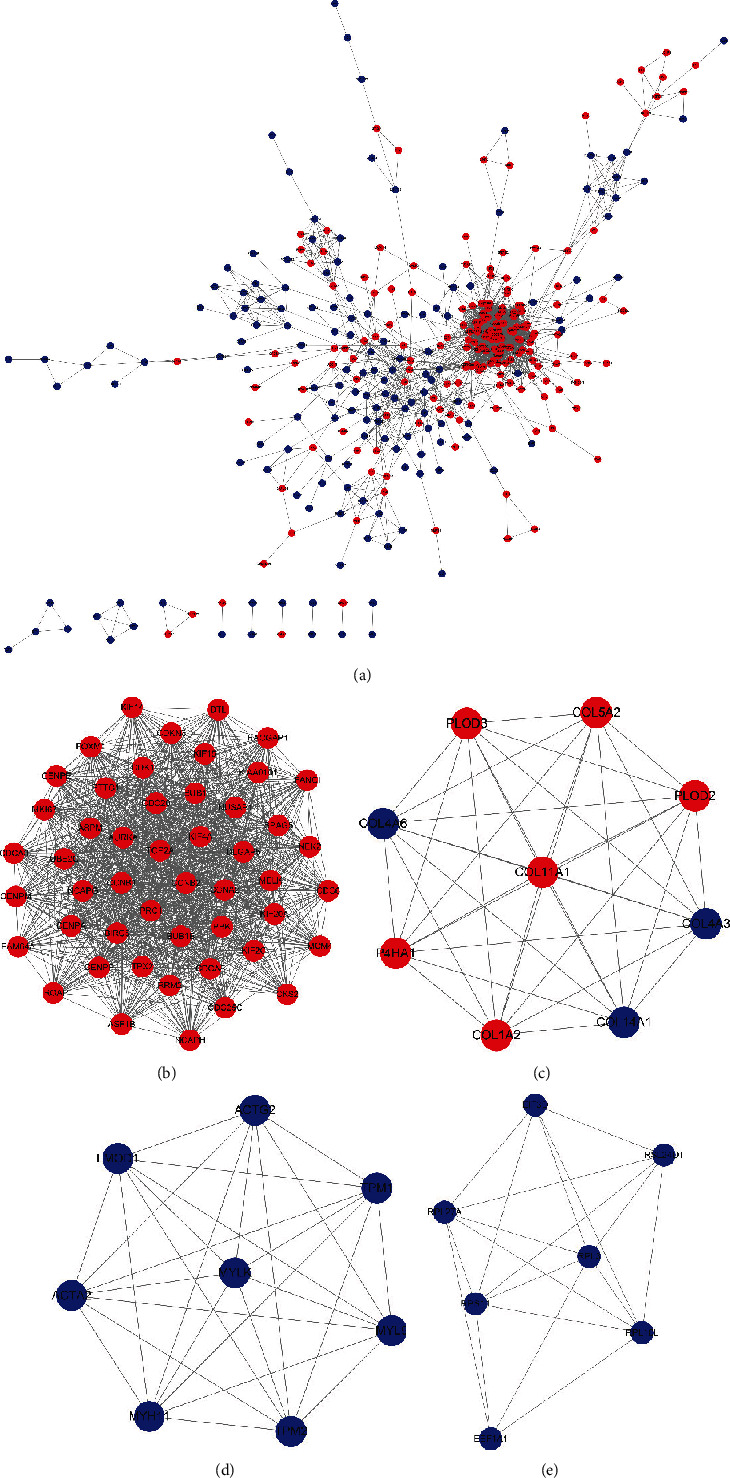
Construction of PPI network and module analysis. (a) The whole PPI networks. (b) PPI network of module 1. (c) PPI network of module 2. (d) PPI network of module 3. (e) PPI network of module 4. PPI: protein-protein interaction. Red represents upregulated genes; blue represents downregulated genes.

To understand the interaction of robust DEGs, the STRING database was applied to construct a PPI network, and the results revealed that 339 nodes and 2362 edges were present in the whole PPI network (Figure 4(a)). The MCODE plug-in was used to identify key modules in the whole PPI network. The top 4 modules are shown in Figures 4(b)–4(e), and the genes in each module are listed in Table 3. Furthermore, pathway enrichment analysis of the top 4 modules was performed in the DAVID database (Supplementary Table S1), and the results indicated that cell cycle, protein digestion and absorption, vascular smooth muscle contraction, and ribosome were the significantly enriched pathways in the individual modules.

Meanwhile, the cytoHubba plug-in, which provides twelve topological analysis methods for ranking nodes in a network by their network features [26], was used to screen hub genes in the whole PPI network. Eventually, sixty-four potential hub genes were screened out according to the maximal clique centrality (MCC) method (Supplementary Table S2), and we selected nine genes that are rarely reported in prostate cancer (NCAPG2, MCM4, KIF18B, CENPM, KNTC1, CENPI, GTSE1, ERCC6L, and FAM64A) for further validation.

### 3.4. Hub Gene Validation in the TCGA and SU2C/PCF Dream Team Databases

**Figure 5:**
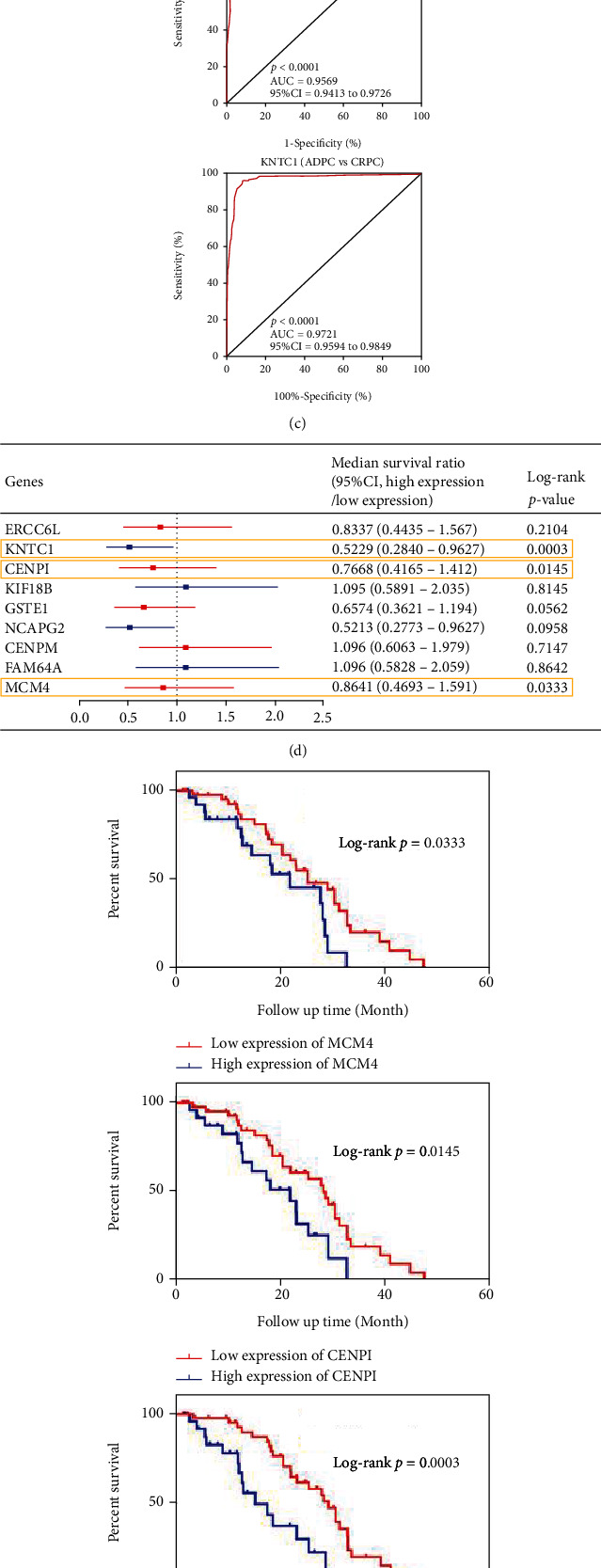
Validation of hub genes in TCGA and SU2C/PCF Dream Team dataset. (a) MCM4, CENPI, and KNTC1 expression variation between ADPC and CRPC. (b) ROC curve analysis of the nine potential hub genes shown with AUC values. (c) ROC curve to assess sensitivity and specificity of MCM4, CENPI, and KNTC1 for diagnosing CRPC. (d) Kaplan-Meier survival analysis of the nine potential hub genes in CRPC shown with median survival ratios. (e) Kaplan-Meier curve indicates that higher expression of MCM4, CENPI, and KNTC1 was correlated with poor survival of CPRC patients. (f) Expression level correlation analysis among the potential hub genes. *P* values were obtained by the Mann–Whitney test, Log-rank test, and Spearman correlation analysis. All data are represented by mean ± SD.

To validate the nine potential hub genes in CRPC, the expression profiles and clinical data of hub genes were downloaded from TCGA (representative for ADPC) and SU2C/PCF Dream Team (representative for CRPC) databases. Subsequently, the data were normalized to the fragments per kilobase of exon model per million mapped fragments (FPKM) value for further analysis. Comparing the expression levels of hub genes in CRPC and ADPC, it was noted that eight of the hub genes were significantly increased in CRPC, and ERCC6L showed a significant decrease (*P* < 0.001, Figure 5(a); Supplementary Figure S1A). We carried out ROC curve analysis and calculated the AUC value. Coincidentally, except for ERCC6L (AUC = 0.6169), the remaining potential hub genes showed high diagnostic value (AUC > 0.85) in distinguishing CRPC from ADPC (Figures 5(b) and 5(c); Supplementary Figure S1B). Afterward, Kaplan–Meier survival analysis and log-rank tests were performed to evaluate the prognostic value of the hub genes. As a result, only MCM4, CENPI, and KNTC1 were found to be associated with poor overall survival in CRPC. The median overall survival (OS) times in the high MCM4, CENPI, and KNTC1 expression groups vs. the low expression groups were 22.14 vs. 25.62, 22.14 vs. 28.88, and 15.37 vs. 29.40 months (log-rank *P* < 0.05), respectively, while the other six potential hub genes showed no significant association with the OS rate in CRPC patients (Figures 5(d) and 5(e); Supplementary Figure S1C). Moreover, coexpression analysis revealed a positive correlation among MCM4, KNTC1, and CENPI (MCM4 vs. KNTC1, *r* = 0.46, *P* < 0.0001; MCM4 vs. CENPI, *r* = 0.54, *P* < 0.0001; KNTC vs. CENPI, *r* = 0.66, *P* < 0.0001) (Figure 5(f)). Of note, these results suggest that MCM4, CENPI, and KNTC1 showed excellent performance as diagnostic and prognostic biomarkers in CRPC.

### 3.5. The Association of Hub Genes with Clinical Features of CRPC in the SU2C/PCF Dream Team Database

**Figure 6:**
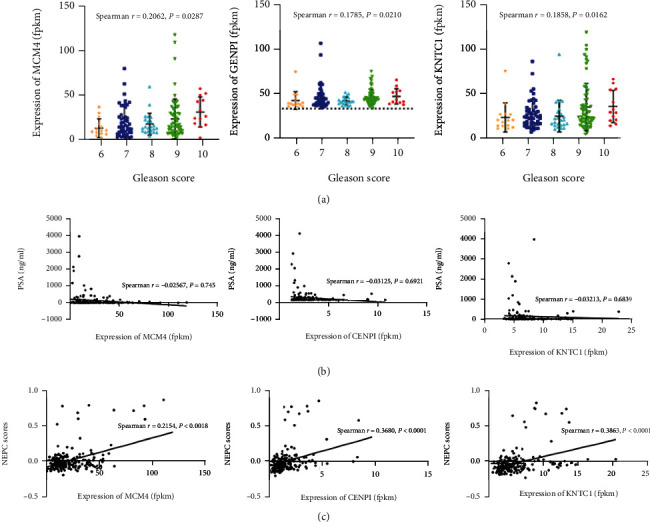
Identification of the association between hub gene and CRPC clinical characteristics. (a) Association of MCM4, CENPI, and KNTC1 expression levels with Gleason scores in CRPC samples. (b) Association of MCM4, CENPI, and KNTC1 expression levels with PSA levels in CRPC patients. (c) Association of MCM4, CENPI, and KNTC1 expression levels with NEPC scores in CRPC patients. *P* values were obtained by Spearman correlation analysis. All data are represented by mean ± SD.

To identify the potential roles of hub genes in CRPC, the association of MCM4, CENPI, and KNTC1 with clinical features of CRPC was evaluated. First, we examined the association of the expression of MCM4, CENPI, and KNTC1 with serum PSA and Gleason score, which is typically used to grade PCa. The results showed that the expression levels of MCM4, KNTC1, and CENPI in CRPC samples were positively correlated with Gleason scores (*P* < 0.05, Figure 6(a)), although the correlation coefficient was low. Additionally, it was noted that none of the hub gene expression levels were associated with serum PSA levels (*P* > 0.5, Figure 6(b)). Finally, we investigated whether the expression of MCM4, CENPI, and KNTC1 was associated with the NEPC score, which was introduced to quantify the degree of CRPC-adenocarcinoma (CRPC-Adeno) conversion to CRPC-neuroendocrine (CRPC-NE), a more aggressive variant of CRPC. Excitingly, the results demonstrated that the expression of all three hub genes was positively correlated with the NEPC score (*P* < 0.01, Figure 6(c)). Based on the above results, the expression levels of these three hub genes may be positively correlated with the malignancy of CRPC.

### 3.6. Hub Genes May Be Involved in CRPC Progression by Regulating Cell Growth and Death-Related Pathways as well as Replication and Repair-Related Pathways: Gene Set Enrichment Analysis (GSEA)

**Figure 7:**
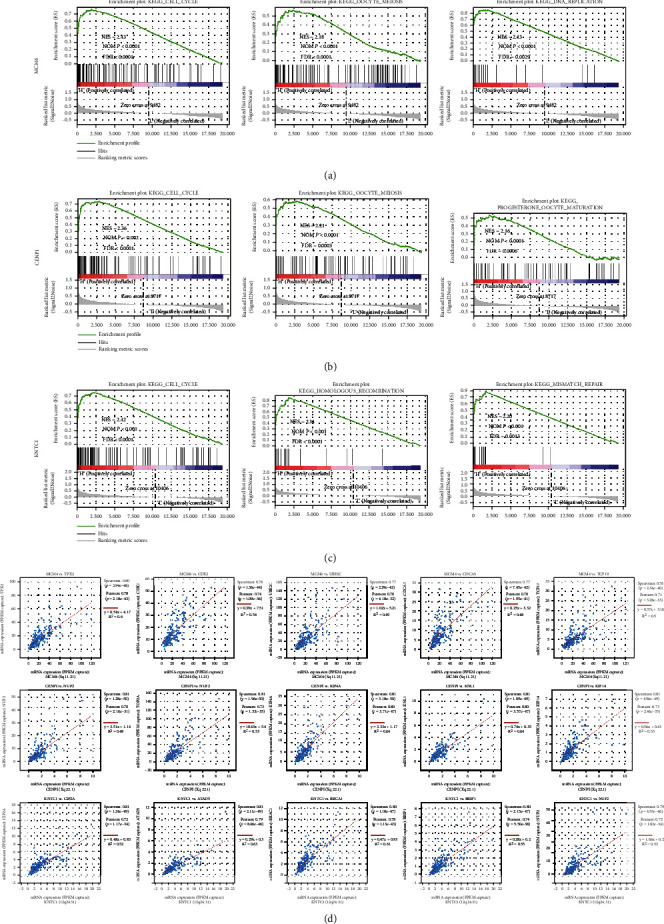
Gene set enrichment analysis (GSEA) of KEGG pathway gene sets in hub genes high-expression versus low-high-expression samples from SU2C/PCF Dream Team dataset. (a–c) Top 3 gene sets (according to GSEA normalized enrichment score) enriched in the high-expression group of each hub gene. (a) MCM4; (b) CENPI; (c) KNTC1. NOM *P* value and FDR are shown in each plot. (d) The top 5 most related genes of each hub gene. *P* values were obtained by Spearman or Pearson correlation analysis.

To determine the potential functional pathways of hub genes, expression-based GSEA was utilized to map KEGG in the SU2C/PCF Dream Team database. The top 3 GSEA pathways were selected according to the normalized enrichment score (NES) ranking [NOM *P* < 0.05, false discovery rate (FDR) < 0.05]. The results showed that high expression of the three hub genes was mainly related to cell growth and death-related pathways and replication and repair-related pathways (Figures 7(a)–7(c)). For cell growth and death-related pathway gene sets, the cell cycle was enriched in the high expression group of all three hub genes, and oocyte meiosis was enriched in the MCM4 and CENPI high expression groups. For replication and repair-related pathway gene sets, DNA replication was enriched in the MCM4 high expression group, and homologous recombination and mismatch repair were enriched in the high KNTC1 expression group. In addition, progesterone-mediated oocyte maturation, an endocrine system-related pathway, was enriched in the high CENPI expression group. A heatmap of gene sets for the top 3 GSEA pathways of each hub gene is shown in Supplementary Figure S2. In terms of the above GSEA results, the enriched pathways of the three hub genes were mainly involved in cell cycle regulation and cell growth regulation. We further performed coexpression analysis to identify the genes most related to the hub genes. Among the top 5 most related genes for each hub gene, chromosomal instability-regulated genes (TPX2, NUF2, TOP2A, BRCA1, and BRIP1, *P* < 0.001, Spearman r > 0.75) and cell cycle-related genes (CDK2, UBE2C, and CDCA5, *P* < 0.001, Spearman *r* > 0.75) accounted for the largest proportion (Figure 7(d)), suggesting that the cell cycle transition regulated by chromosomal instability may be a potential mechanism affecting CRPC progression.

### 3.7. The Hub Genes Govern the Cell Cycle in CRPC and Promote Cell Proliferation

**Figure 8:**
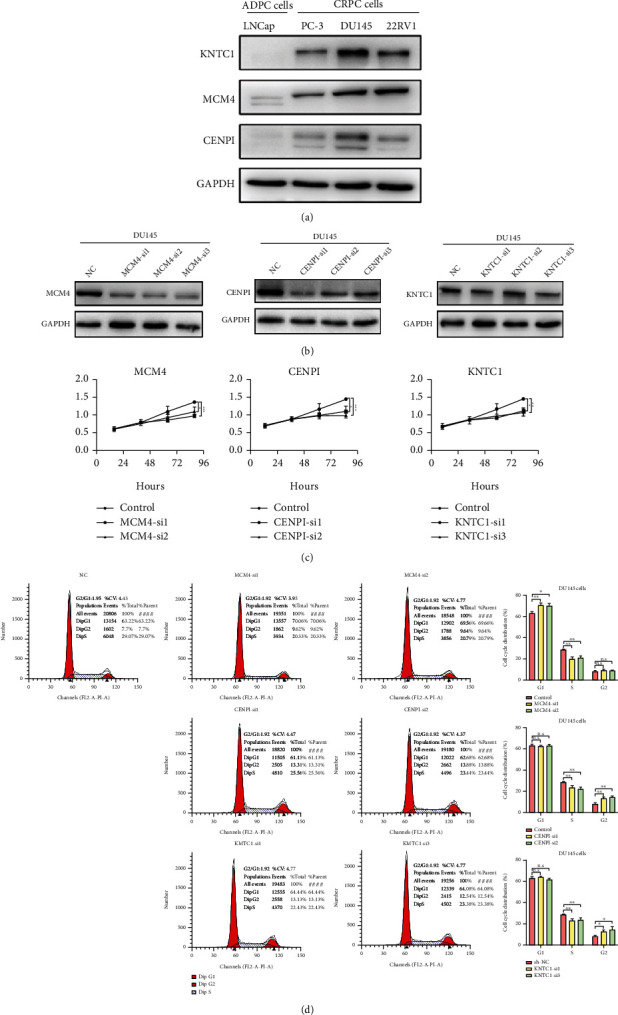
Hub genes are upregulated in the CRPC cell line and promote cell proliferation through governing cell cycle. (a) Protein expression levels of hub genes in PCa cell line. (b) MCM4, CENPI, and KNTC1 protein levels in indicated cells transfected with target siRNAs. (c) CCK-8 assay evaluates cell viability of CRPC cells after transfection with target siRNAs. (d) Cell cycle distribution analysis of CRPC cells after transfection with target siRNAs using flow cytometry. *P* values were obtained by Student's *t*-test. Bars represent the means ± SD of three replicates. ^∗^*P* ≤ 0.05, ^∗∗^*P* ≤ 0.01, and ^∗∗∗^*P* ≤ 0.001.

To further confirm the hub genes validated in the TCGA and SU2C/PCF Dream Team databases and clarify their biological functions in CRPC, we investigated hub gene expression levels in four PCa cell lines, representing ADPC (LNCaP) and CRPC (PC3, DU145, and 22RV1). As expected, the expression levels of the three hub genes were significantly increased in CRPC cells compared to ADPC cells (Figure 8(a)). CCK-8 assays were used to estimate hub gene effects on CRPC cell proliferation, and the results revealed that inhibition of hub genes suppressed CRPC cell growth (Figures 8(b) and 8(c)). Based on the GSEA results, the three hub genes may play vital roles in controlling the cell cycle. Thus, flow cytometry was carried out to analyze the changes in cell cycle distribution after the expression of the hub genes was suppressed. The results suggested that MCM4 silencing led to CRPC cell arrest in G1 phase, while CENPI and KNTC1 silencing caused CRPC cells to arrest in G2 phase (Figure 8(d)), confirming the vital roles of hub genes in regulating cell cycle transition in CRPC. In addition, MCM4/6/7 helicase inhibitor heliquinomycin shows a similar effect with MCM4 siRNAs in hindering CRPC cell growth (Supplementary Figure S3). Regrettably, no inhibitors of CENPI and KNTC1 have been developed on the market at present. Overall, we provide evidence that the three hub genes screened out by our comprehensive bioinformatics analysis are expressed at a higher level in CRPC than in ADPC and may play critical roles in regulating the cell cycle to influence CRPC progression.

## 4. Discussion

The conversion of ADPC to CRPC remains a rigorous challenge for prostate cancer treatment [2]. Therefore, exploration of hub genes involved in CRPC progression is of profound significance in providing new insights into CRPC therapeutic strategies. At present, although numerous individual studies have used gene expression arrays and RNA-seq to discover biomarkers and therapeutic targets for CRPC [28, 29], considering differences in xenograft-derived tumor tissues, sample sizes, and technology platforms, the results among different studies are always inconsistent [9–11]. In this study, to overcome the above shortcomings, we only selected GEO expression profiles derived from patient tumor tissue (including CRPC and ADPC) and then applied the RRA method to integrate different expression profiles, searching for robust DEGs among different studies. Eventually, we screened 261 upregulated and 266 downregulated robust DEGs.

Among the top 20 significantly upregulated and downregulated robust DEGs, several genes have been reported to participate in CRPC progression. For example, in CRPC, the AR coactivators SRC1, SRC3, p300, and MED1 bind to the UBE2C enhancer, resulting in overexpression and activation of UBE2C, thereby inactivating the M-phase checkpoint, governing the cell cycle, and promoting CRPC cell proliferation [30, 31]. FOXM1, another top 20 significantly upregulated robust DEG, is considered a master regulator of enzalutamide-resistant (ENZ^R^) CRPC, and targeting FOXM1 reduces cell growth and stemness in ENZ^R^ CRPC in vitro and in vivo [32]. In addition, among the top 20 significantly downregulated robust DEGs, DPP4 was identified as an AR-stimulated tumor suppressor gene that is downregulated in the progression of CRPC transformation, leading to CRPC cell growth [33]. Given the above published research, the reliability of robust DEGs screened by the RRA method was confirmed, and several new insights were provided for us to investigate the potential mechanism of CRPC.

To deeply understand the functions of robust DEGs in CRPC, GO and KEGG analyses were performed using the DAVID database. Among the upregulated robust DEGs, several GO terms, such as cell division, protein binding, and ATP binding, were mainly enriched and confirmed to be involved in the progression of CRPC [34–36]. Furthermore, KEGG enrichment of the upregulated DEGs was mainly distributed in the cell cycle, pathway in cancer and PI3K-Akt signaling pathway. To the best of our knowledge, most of the genes that were identified as robust DEGs are widely involved in cell cycle governance in CRPC cells [37–39]. The PI3K-Akt signaling pathway is a key oncogenic pathway and plays a pivotal role in cancer progression, drug resistance, and treatment in various cancer types [40, 41], and its constitutive activation due to loss of PTEN in most advanced prostate cancer cases contributes to resistance to androgen deprivation therapy [42, 43]. Therefore, targeting PI3K-Akt is considered a promising approach for the treatment of CRPC [44, 45]. Unsurprisingly, Marques and colleagues revealed that using PI3K-Akt inhibitors combined with androgen deprivation improves the treatment efficacy in prostate cancer [46]. Among the downregulated robust DEGs, positive/negative regulation of transcription from the RNA polymerase II promoter, extracellular exosome, and sequence-specific DNA binding were the main enrichment GO terms. Interestingly, it has been reported that exosomes play a promotive role in mediating multidrug resistance in most cancer types, including the induction of CRPC neuroendocrine differentiation by delivering adipocyte differentiation-related proteins [47, 48]. KEGG enrichment analysis showed that downregulated DEGs were concentrated in pathways in cancer, HTLV-1 infection, and focal adhesions. Notably, focal adhesion kinase phosphorylation mediates docetaxel resistance in CRPC [49]. Given the robust DEG functional enrichment results, there are enough reasons to believe that these DEGs may be closely related to CRPC progression and drug resistance.

The robust DEGs were used to construct a PPI network, and key modules were identified with the MCODE plug-in of Cytoscape software. Interestingly, pathway enrichment analysis of the key modules revealed that robust DEGs in the significant module were mostly enriched in the cell cycle, which is consistent with the KEGG pathway enrichment results for upregulated robust DEGs. The maximal clique centrality algorithm (MCC) was employed to screen hub genes in the whole PPI network. According to the MCC value, sixty-four genes were screened out, and we selected nine genes rarely reported in PCa for further validation (NCAPG2, MCM4, KIF18B, CENPM, KNTC1, CENPI, GTSE1, ERCC6L, and FAM64A). Through a series of validations of expression variation, ROC curve analyses, and K-M plots in TCGA and the SU2C/PCF Dream Team database, we identified three hub genes, namely, MCM4, CEPNI, and KNTC1, which are of high diagnostic and prognostic value for CRPC. Unexpectedly, the three genes have no significant correlation with serum PSA in CRPC. However, the expression levels of the three hub genes were found to be positively correlated with NEPC scores, which represent the degree of CRPC-Adeno transformation into CRPC-NE (a more aggressive CRPC variant) [50]. Some reports have emphasized that CRPC-NE does not secrete PSA, manifests as low PSA progression, and is characterized by unresponsiveness to hormone therapy [51–53], supporting the above arguments. Meanwhile, we evaluated the relationship between NEPC scores and Gleason scores, and the results revealed that no significant correlation existed between the two clinical features in CRPC (Spearman *r* = 0.1090, *P* = 0.0799, Supplementary Figure S1D), although the expression of the three hub genes showed a certain correlation with the Gleason score. Coincidentally, Krauss and colleagues emphasized that no significant difference in neuroendocrine differentiation exist between patients with different Gleason scores [54]. In addition, the three hub genes were also verified in ADPC and CRPC at the cellular level. These conclusions provide evidence that the three hub genes could serve as potential diagnostic and prognostic biomarkers of CRPC and strengthen the argument that the three hub genes may play vital roles in CRPC progression.

Chromosomal instability (CIN), also known as genomic instability, is a hallmark of human cancer. It is associated with metastasis, therapeutic resistance, and poor prognosis [55]. It has been reported that MCM4 and CENPI are involved in mediating chromosomal stability to influence the cell cycle and that KNTC1 knockdown suppresses cell viability and induces apoptosis in esophageal squamous cell carcinoma [56–58]. In coexpression analysis, MCM4 was significantly associated with TPX2, which is a microtubule-associated protein that can activate the cell cycle kinase Aurora A and regulate the mitotic spindle, affecting chromosomal stability [59]. In addition, MCM4 was markedly related to cell cycle-regulating genes, such as CDK2, UBE2C, and CDCA2 [30, 60, 61]. NUF2, a component of the kinetochore NDC80 complex, plays an integral role in regulating the binding of the centromere and spindle microtubules to achieve the correct separation of chromosomes. Due to the irreversibility of separation, any error can cause cell death or chromosomal instability [62, 63]. It is worth noting that the NUF2 expression level was positively correlated with the expression levels of CENPI and KNTC1. BRCA1 is a major homologous recombination-mediated repair (HRR) support protein that can work in conjunction with certain chaperone proteins (including RAD51, CTIP, and BRIP1), and it promotes the proper HRR process to regulate the stability of chromosomes [64]. Its expression level also showed a clear correlation with the expression level of KNTC1. GSEA results indicated that variations in the expression of the three hub genes may influence cell growth and death-related pathways and replication and repair-related pathways, such as the cell cycle, oocyte meiosis, homologous recombination, DNA replication, progesterone-mediated oocyte maturation, and mismatch repair. In vitro experimental results support the thesis that the three hub genes are involved in governing the cell cycle and promoting CRPC cell proliferation. Combining bioinformatics analysis and in vitro experiments, the three hub genes may promote CRPC progression by affecting chromosomal instability and could be potential therapeutic targets.

Overall, based on retrospective analysis of available public databases, this study provides a new understanding of potential hub genes involved in CRPC progression. The three hub genes identified accurately distinguished CRPC patients from ADPC patients, and their high expression was found to be correlated with poor prognosis. Moreover, the three hub genes have important biological functions and clinical value in CRPC. Nevertheless, our study still has some limitations. On the one hand, our model is based on retrospective analysis and needs to be validated by primary data from prospective studies. On the other hand, this study is mainly based on bioinformatics analysis, but the functional mechanisms and interactions of genes are complex, and more in vitro and in vivo experiments are needed for verification and evaluation. Moreover, although the three hub genes showed excellent diagnostic and prognostic value in available public databases, this result is based on bioinformatics predictions and requires further validation in prospective cohorts.

## 5. Conclusion

Our research imaginatively integrated different datasets and a series of bioinformatics tools to identify potential hub genes. We finally screened three hub genes (MCM4, CENPI, and KNTC1) that showed high diagnostic and prognostic value in CRPC. Given that most reported CRPC biomarkers are associated with genomic aberrations, the biomarkers identified in this study enrich the types of biomarkers associated with transcriptome expression changes. Furthermore, the prognostic value provided by the three hub genes is exactly what the existing biomarkers lack. In addition, we revealed the underlying biological functions and regulatory networks of the three hub genes in CRPC, which contributes to a deeper understanding of the molecular mechanisms involved in CRPC progression. In total, the three hub genes identified in this study could serve as candidate diagnostic and prognostic biomarkers of CRPC and may provide potential preventive and therapeutic targets for CRPC. However, further efforts should be made to fully reveal the potential mechanisms of hub genes involved in CRPC progression and to validate their feasibility as diagnostic and/or prognostic markers in clinical practice.

## Figures and Tables

**Table 1:** The list of the target sequence for siRNAs.

Genes	Target sequences
MCM4-si1	GCAGAAGAUAUAGUGGCAATT
MCM4-si2	GCAUGGCACUCAUCCACAATT
MCM4-si3	GCUGCCUCAUACUUUAUUATT
CENPI-si1	GCUUAUUCCCUCCAUCUUATT
CENPI-si2	GCUAAGGACUUUGGUAAAUTT
CENPI-si3	CCUCCUGUCUCGUCCAAUUTT
KNTC1-si1	GCUGUAAACACACGGAUAUTT
KNTC1-si2	CCAACUUCCUGGAUACCAUTT
KNTC1-si3	GCUGGUAUUUGGACUAUUUTT

**Table 2:** Characteristics of the included datasets.

Dataset ID	Country	Number of samples	GPL ID	Number of rows per platform
GSE3325	USA	6CRPC 6ADPC	GPL570	23520
GSE32269	USA	29CRPC 22ADPC	GPL96	22282
GSE35988	USA	27CRPC 49ADPC	GPL6480	19596

Note: GSE: Gene Expression Omnibus series; GPL: Gene Expression Omnibus platform; CRPC: castration-resistant prostate cancer; ADPC: androgen-dependent prostate cancer.

**Table 3:** The enriched genes list of top 4 key modules.

Modules	Nodes	Edges	Genes
Module 1	49	1078	MCM4, KIF4A, DLGAP5, SPAG5, PTTG1, MELK, RACGAP1, KIF20A, TROAP, KIAA0101 DTL, CENPF, ASPM, FANCI, CENPM, CDKN3, PRC1, CENPA, CDK1, AURKA, KIF2C FAM64A, CDCA8, CDCA3, CDC20, BUB1, NUSAP1, TPX2, CDC6, BUB1B, CCNA2 FOXM1, NEK2, CKS2, CDC25C, CCNB1, PBK, ASF1B, MKI67, CCNB2, TOP2A, KIF14 BIRC5, NCAPG, NCAPH, KIF15, RRM2, UBE2C, CENPE
Module 2	9	36	COL11A1, P4HA1, COL4A6, PLOD2, PLOD3, COL4A3, COL14A1, COL5A2, COL1A2
Module 3	8	28	MYLK, MYL9, TPM1, LMOD1, ACTG2, ACTA2, TPM2, MYH11
Module 4	7	19	RPL27A, RPS11, EIF3D, RPL3, RPL10L, EEF1A1, RSL24D1

## Data Availability

All data generated or analyzed during this study are included in this published article.

## Abbreviations

ADPC: Androgen-dependent prostate cancer
AR: Androgen receptor
AUC: Area under the ROC curve
BP: Biological processes
CC: Cell component
CIN: Chromosomal instability
CRPC: Castration-resistant prostate cancer
DAVID: Database for Annotation, Visualization and Integrated Discovery
DEGs: Differentially expressed genes
FDR: False discovery rate
FPKM: Fragments per kilobase of exon per million reads mapped
GEO: Gene Expression Omnibus database
GO: Gene Ontology
GSEA: Gene set enrichment analysis
HRR: Homologous recombination-mediated repair
KEGG: Kyoto Encyclopedia of Genes and Gene Ontology
MCC: Maximal clique centrality
MF: Molecular function
NEPC: Neuroendocrine prostate cancer
NES: Normalized enrichment score
NOM: Nominal
OS: Overall survival
PPI: Protein-protein interaction network
PSA: Prostate-specific antigen
RRA: Robust rank aggregation
ROC: Receiver-operating characteristic
SU2C/PCF: SU2C-Prostate Cancer Foundation Prostate Dream Team
TCGA: The Cancer Genome Atlas.

## Supplementary Materials

Supplementary Materials Figure S1: validation of the other six potential hub genes in TCGA and SU2C/PCF Dream Team dataset. (A) FAM64A, CENPM, NCAPG2, GSTE1, KIF18B, and ERCC6L expression differences between ADPC and CRPC. (B) ROC curves of FAM64A, CENPM, NCAPG2, GSTE1, KIF18B, and ERCC6L for diagnosing CRPC. (C) Kaplan-Meier survival analysis of FAM64A, CENPM, NCAPG2, GSTE1, KIF18B, and ERCC6L in CRPC. (D) The correlation analysis between NEPC score and Gleason score. *P* values were obtained by the Mann–Whitney test, log-rank test, and Pearson correlation analysis. Figure S2: the heatmap of gene sets for the top3 GSEA pathway of three hub genes. (A) The heatmap of gene sets for the top3 GSEA pathway of MCM4. (B) The heatmap of gene sets for the top3 GSEA pathway of CENPI. (C) The heatmap of gene sets for the top3 GSEA pathway of KNTC1. Figure S3: the MCM4/6/7 helicase inhibitor heliquinomycin suppresses DU145 cell proliferation by inducing cell cycle arrest in G1 phase. (A) CCK-8 assay evaluates the cell viability of DU145 cells after treatment with heliquinomycin. (B) Cell cycle distribution analysis of DU145 cells after treatment with heliquinomycin using flow cytometry. *P* values were obtained by Student's *t*-test. Bars represent the means ± SD of three replicates. ^∗^*P* ≤ 0.05, ^∗∗^*P* ≤ 0.01, and ^∗∗∗^*P* ≤ 0.001. Table S1: the most enriched pathways in top4 modules. Table S2: the list of top sixty-four ranking nodes in the whole PPI network according to the maximal clique centrality (MCC) method.
