# Supplementary material for: Identification and Validation of Three Hub Genes Involved in Cell Proliferation and Prognosis of Castration-Resistant Prostate Cancer
Source: Oxid Med Cell Longev. 2022 Aug 17;2022:8761112. doi: 10.1155/2022/8761112 (PMC9402298; doi:10.1155/2022/8761112)
Supplement: Supplementary Materials — Figure S1: validation of the other six potential hub genes in TCGA and SU2C/PCF Dream Team dataset. (A) FAM64A, CENPM, NCAPG2, GSTE1, KIF18B, and ERCC6L expression differences between ADPC and CRPC. (B) ROC curves of FAM64A, CENPM, NCAPG2, GSTE1, KIF18B, and ERCC6L for diagnosing CRPC. (C) Kaplan-Meier survival analysis of FAM64A, CENPM, NCAPG2, GSTE1, KIF18B, and ERCC6L in CRPC. (D) The correlation analysis between NEPC score and Gleason score. P values were obtained by the Mann–Whitney test, log-rank test, and Pearson correlation analysis. Figure S2: the heatmap of gene sets for the top3 GSEA pathway of three hub genes. (A) The heatmap of gene sets for the top3 GSEA pathway of MCM4. (B) The heatmap of gene sets for the top3 GSEA pathway of CENPI. (C) The heatmap of gene sets for the top3 GSEA pathway of KNTC1. Figure S3: the MCM4/6/7 helicase inhibitor heliquinomycin suppresses DU145 cell proliferation by inducing cell cycle arrest in G1 phase. (A) CCK-8 assay evaluates the cell viability of DU145 cells after treatment with heliquinomycin. (B) Cell cycle distribution analysis of DU145 cells after treatment with heliquinomycin using flow cytometry. P values were obtained by Student's t-test. Bars represent the means ± SD of three replicates. ∗P ≤ 0.05, ∗∗P ≤ 0.01, and ∗∗∗P ≤ 0.001. Table S1: the most enriched pathways in top4 modules. Table S2: the list of top sixty-four ranking nodes in the whole PPI network according to the maximal clique centrality (MCC) method. [file 8761112.f1.zip › Supplementary Figure 1.pdf]

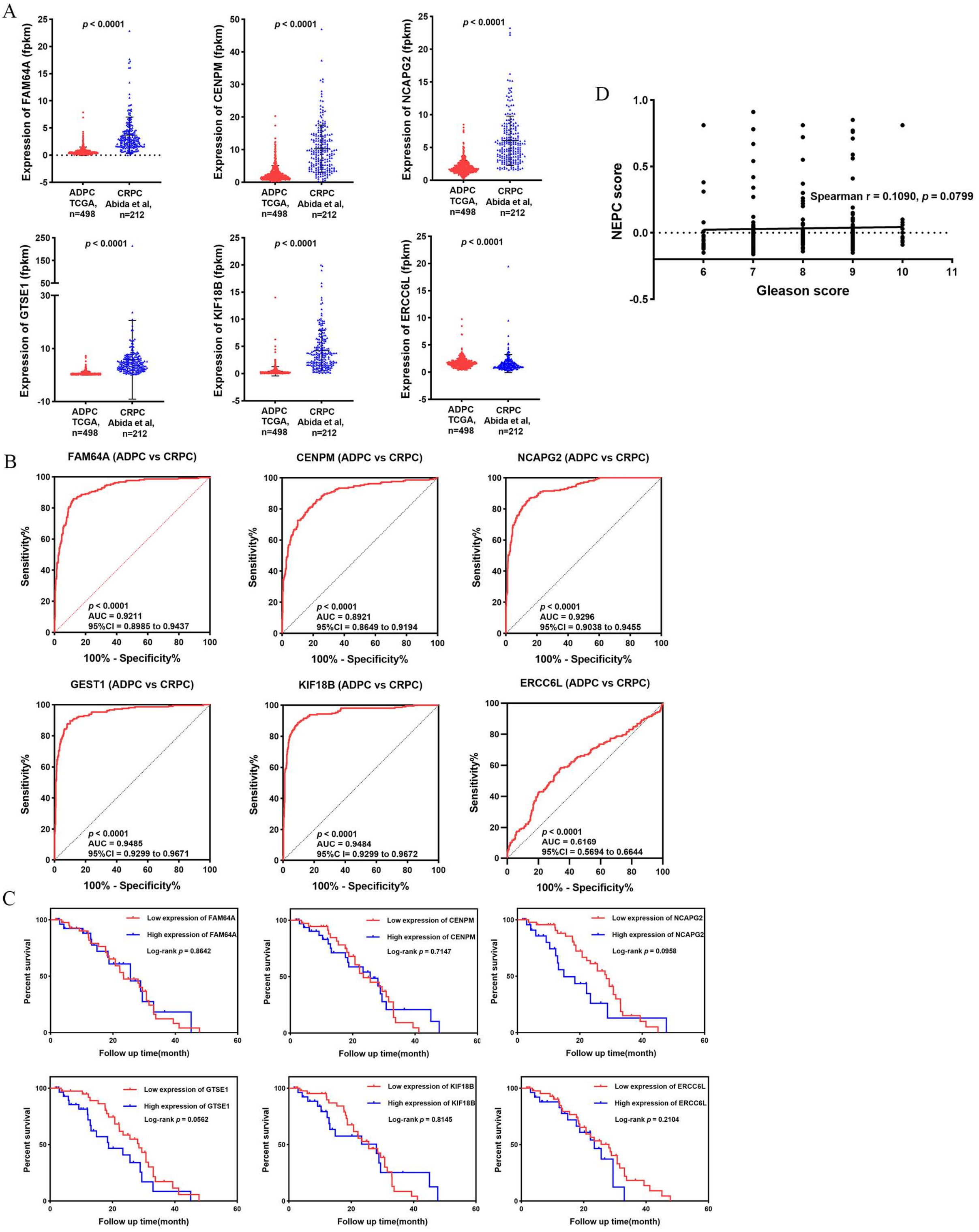
A
p < 0.0001
Expression of FAM64A (fpkm)
Expression of CENPM (fpkm)
Expression of NCAPG2 (fpkm)
Expression of GTSE1 (fpkm)
Expression of KIF18B (fpkm)
Expression of ERCC6L (fpkm)
ADPC TCGA, n=498
CRPC Abida et al, n=212
D
NEPC score
Gleason score
Spearman r = 0.1090, p = 0.0799
B
FAM64A (ADPC vs CRPC)
p < 0.0001
AUC = 0.9211
95%CI = 0.8985 to 0.9437
CENPM (ADPC vs CRPC)
p < 0.0001
AUC = 0.8921
95%CI = 0.8649 to 0.9194
NCAPG2 (ADPC vs CRPC)
p < 0.0001
AUC = 0.9296
95%CI = 0.9038 to 0.9455
GEST1 (ADPC vs CRPC)
p < 0.0001
AUC = 0.9485
95%CI = 0.9299 to 0.9671
KIF18B (ADPC vs CRPC)
p < 0.0001
AUC = 0.9484
95%C I= 0.9299 to 0.9672
ERCC6L (ADPC vs CRPC)
p < 0.0001
AUC = 0.6169
95%CI = 0.5694 to 0.6644
Sensitivity%
100% - Specificity%
C
Low expression of FAM64A
High expression of FAM64A
Log-rank p = 0.8642
Low expression of CENPM
High expression of CENPM
Log-rank p = 0.7147
Low expression of NCAPG2
High expression of NCAPG2
Log-rank p = 0.0958
Low expression of GTSE1
High expression of GTSE1
Log-rank p = 0.0562
Low expression of KIF18B
High expression of KIF18B
Log-rank p = 0.8145
Low expression of ERCC6L
High expression of ERCC6L
Log-rank p = 0.2104
Percent survival
Follow up time(month)

Figure S1. Validation of the other six potential hub genes in TCGA and SU2C/PCF Dream Team dataset. (A) FAM64A, CENPM, NCAPG2, GSTE1, KIF18B, and ERCC6L expression differences between ADPC and CRPC. **(B)** ROC curves of FAM64A, CENPM, NCAPG2, GSTE1, KIF18B, and ERCC6L for diagnosing CRPC. **(C)** Kaplan-Meier survival analysis of FAM64A, CENPM, NCAPG2, GSTE1, KIF18B, and ERCC6L in CRPC. **(D)** The correlation analysis between NEPC score and Gleason score. *P*-values were obtained by the Mann-Whitney test, Log-rank test, and Pearson correlation analysis.
