# Supplementary material for: Identification and Validation of Three Hub Genes Involved in Cell Proliferation and Prognosis of Castration-Resistant Prostate Cancer
Source: Oxid Med Cell Longev. 2022 Aug 17;2022:8761112. doi: 10.1155/2022/8761112 (PMC9402298; doi:10.1155/2022/8761112)
Supplement: Supplementary Materials — Figure S1: validation of the other six potential hub genes in TCGA and SU2C/PCF Dream Team dataset. (A) FAM64A, CENPM, NCAPG2, GSTE1, KIF18B, and ERCC6L expression differences between ADPC and CRPC. (B) ROC curves of FAM64A, CENPM, NCAPG2, GSTE1, KIF18B, and ERCC6L for diagnosing CRPC. (C) Kaplan-Meier survival analysis of FAM64A, CENPM, NCAPG2, GSTE1, KIF18B, and ERCC6L in CRPC. (D) The correlation analysis between NEPC score and Gleason score. P values were obtained by the Mann–Whitney test, log-rank test, and Pearson correlation analysis. Figure S2: the heatmap of gene sets for the top3 GSEA pathway of three hub genes. (A) The heatmap of gene sets for the top3 GSEA pathway of MCM4. (B) The heatmap of gene sets for the top3 GSEA pathway of CENPI. (C) The heatmap of gene sets for the top3 GSEA pathway of KNTC1. Figure S3: the MCM4/6/7 helicase inhibitor heliquinomycin suppresses DU145 cell proliferation by inducing cell cycle arrest in G1 phase. (A) CCK-8 assay evaluates the cell viability of DU145 cells after treatment with heliquinomycin. (B) Cell cycle distribution analysis of DU145 cells after treatment with heliquinomycin using flow cytometry. P values were obtained by Student's t-test. Bars represent the means ± SD of three replicates. ∗P ≤ 0.05, ∗∗P ≤ 0.01, and ∗∗∗P ≤ 0.001. Table S1: the most enriched pathways in top4 modules. Table S2: the list of top sixty-four ranking nodes in the whole PPI network according to the maximal clique centrality (MCC) method. [file 8761112.f1.zip › Supplementary Figure 2.pdf]

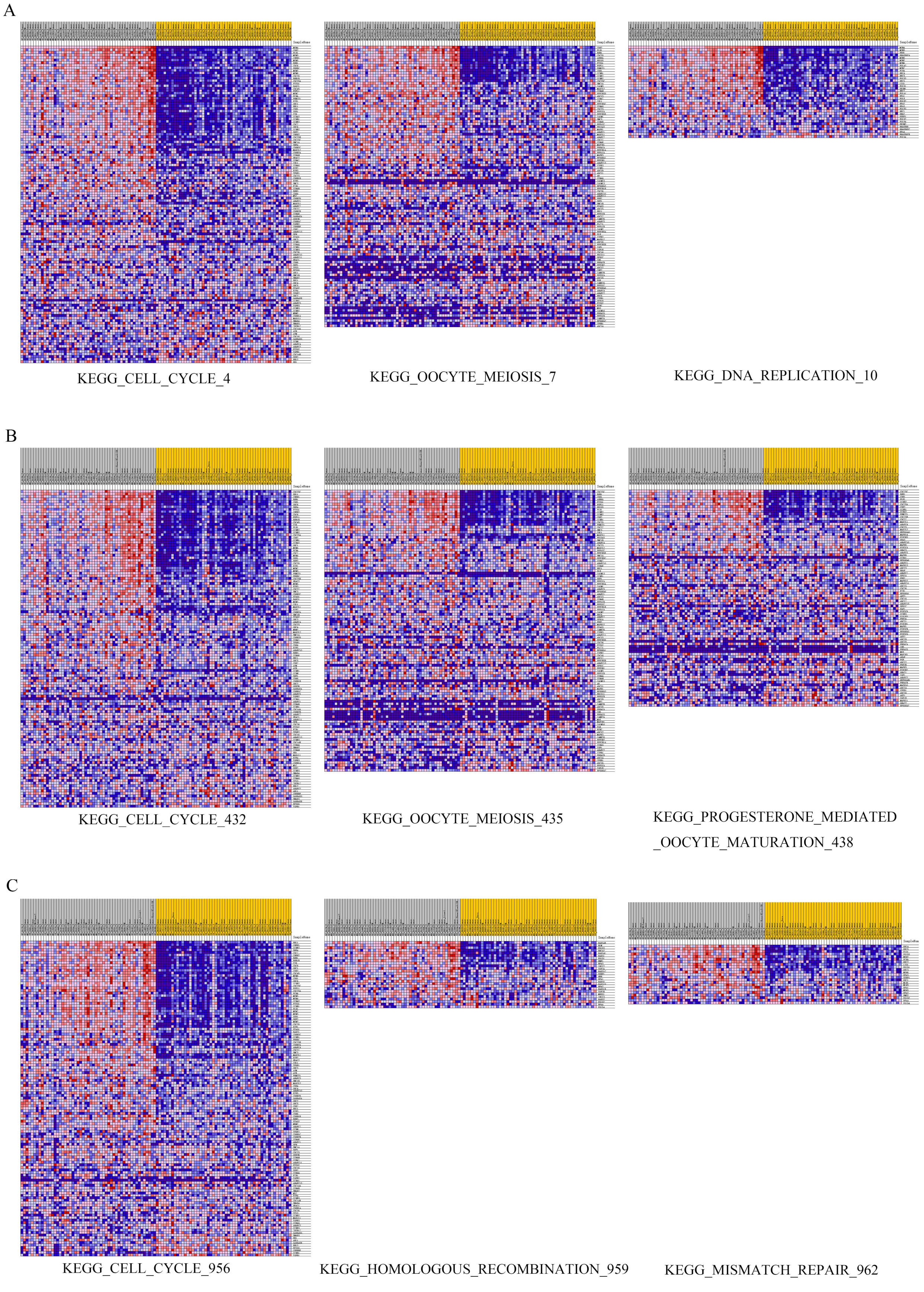
A
KEGG_CELL_CYCLE_4
KEGG_OOCYTE_MEIOSIS_7
KEGG_DNA_REPLICATION_10
B
KEGG_CELL_CYCLE_432
KEGG_OOCYTE_MEIOSIS_435
KEGG_PROGESTERONE_MEDIATED
_OOCYTE_MATURATION_438
C
KEGG_CELL_CYCLE_956
KEGG_HOMOLOGOUS_RECOMBINATION_959
KEGG_MISMATCH_REPAIR_962

Figure S2. The heatmap of gene sets for the top3 GSEA pathway of three hub genes. (A) The heatmap of gene sets for the top3 GSEA pathway of MCM4. **(B)** The heatmap of gene sets for the top3 GSEA pathway of CENPI. **(C)** The heatmap of gene sets for the top3 GSEA pathway of KNTC1.
