# Supplementary material for: Identification and Validation of Three Hub Genes Involved in Cell Proliferation and Prognosis of Castration-Resistant Prostate Cancer
Source: Oxid Med Cell Longev. 2022 Aug 17;2022:8761112. doi: 10.1155/2022/8761112 (PMC9402298; doi:10.1155/2022/8761112)
Supplement: Supplementary Materials — Figure S1: validation of the other six potential hub genes in TCGA and SU2C/PCF Dream Team dataset. (A) FAM64A, CENPM, NCAPG2, GSTE1, KIF18B, and ERCC6L expression differences between ADPC and CRPC. (B) ROC curves of FAM64A, CENPM, NCAPG2, GSTE1, KIF18B, and ERCC6L for diagnosing CRPC. (C) Kaplan-Meier survival analysis of FAM64A, CENPM, NCAPG2, GSTE1, KIF18B, and ERCC6L in CRPC. (D) The correlation analysis between NEPC score and Gleason score. P values were obtained by the Mann–Whitney test, log-rank test, and Pearson correlation analysis. Figure S2: the heatmap of gene sets for the top3 GSEA pathway of three hub genes. (A) The heatmap of gene sets for the top3 GSEA pathway of MCM4. (B) The heatmap of gene sets for the top3 GSEA pathway of CENPI. (C) The heatmap of gene sets for the top3 GSEA pathway of KNTC1. Figure S3: the MCM4/6/7 helicase inhibitor heliquinomycin suppresses DU145 cell proliferation by inducing cell cycle arrest in G1 phase. (A) CCK-8 assay evaluates the cell viability of DU145 cells after treatment with heliquinomycin. (B) Cell cycle distribution analysis of DU145 cells after treatment with heliquinomycin using flow cytometry. P values were obtained by Student's t-test. Bars represent the means ± SD of three replicates. ∗P ≤ 0.05, ∗∗P ≤ 0.01, and ∗∗∗P ≤ 0.001. Table S1: the most enriched pathways in top4 modules. Table S2: the list of top sixty-four ranking nodes in the whole PPI network according to the maximal clique centrality (MCC) method. [file 8761112.f1.zip › Supplementary Table 1.pdf]

Supplementary Table 1: The most enriched pathways in top4 modules

**Module 1 KEGG enrichment**						
**Term**	**Genes**	**Fold enrichment**	***P*-value**	**Bonferroni**	**Benjamini**	**FDR**
hsa04110: Cell cycle	CCNB1, CDK1, CDC6, CCNB2, BUB1, BUB1B, CDC20, PTTG1, CDC25C, CCNA2, MCM4	35.89611	1.84E-14	4.42E-13	4.42E-13	1.48E-11
hsa04114: Oocyte meiosis	CCNB1, CDK1, CCNB2, BUB1, AURKA, CDC20, PTTG1, CDC25C	29.16375	2.39E-09	5.74E-08	2.87E-08	1.92E-06
hsa04914: Progesterone-mediated oocyte maturation	CCNB1, CDK1, CCNB2, BUB1, CDC25C, CCNA2	27.90669	1.13E-06	2.71E-05	9.03E-06	9.05E-04
**Module 2 KEGG enrichment**						
**Term**	**Genes**	**Fold enrichment**	***P*-value**	**Bonferroni**	**Benjamini**	**FDR**
hsa04974: Protein digestion and absorption	COL4A3, COL14A1, COL1A2, COL11A1, COL5A2, COL4A6	52.11364	1.66E-08	1.99E-07	1.99E-07	1.07E-05
hsa04512: ECM-receptor interaction	COL4A3, COL1A2, COL11A1, COL5A2, COL4A6	43.9272	1.61E-06	1.93E-05	9.65E-06	0.001034
hsa05146: Amoebiasis	COL4A3, COL1A2, COL11A1, COL5A2, COL4A6	36.05346	3.56E-06	4.27E-05	1.42E-05	0.002288
hsa04510: Focal adhesion	COL4A3, COL1A2, COL11A1, COL5A2, COL4A6	18.55178	4.98E-05	5.97E-04	1.49E-04	0.032008
**Module 3 KEGG enrichment**						
**Term**	**Genes**	**Fold enrichment**	***P*-value**	**Bonferroni**	**Benjamini**	**FDR**
hsa04270: Vascular smooth muscle contraction	ACTG2, ACTA2, MYLK, MYL9	33.59707	9.24E-05	0.001477	0.001477	0.065601
**Module 3 KEGG enrichment**						
**Term**	**Genes**	**Fold enrichment**	***P*-value**	**Bonferroni**	**Benjamini**	**FDR**
hsa03010: Ribosome	RPL10L, RPL27A, RPL3, RPS11, RSL24D1	36.1292	2.13E-06	6.38E-06	6.38E-06	6.70E-04
