# Supplementary material for: Identification and Validation of Three Hub Genes Involved in Cell Proliferation and Prognosis of Castration-Resistant Prostate Cancer
Source: Oxid Med Cell Longev. 2022 Aug 17;2022:8761112. doi: 10.1155/2022/8761112 (PMC9402298; doi:10.1155/2022/8761112)
Supplement: Supplementary Materials — Figure S1: validation of the other six potential hub genes in TCGA and SU2C/PCF Dream Team dataset. (A) FAM64A, CENPM, NCAPG2, GSTE1, KIF18B, and ERCC6L expression differences between ADPC and CRPC. (B) ROC curves of FAM64A, CENPM, NCAPG2, GSTE1, KIF18B, and ERCC6L for diagnosing CRPC. (C) Kaplan-Meier survival analysis of FAM64A, CENPM, NCAPG2, GSTE1, KIF18B, and ERCC6L in CRPC. (D) The correlation analysis between NEPC score and Gleason score. P values were obtained by the Mann–Whitney test, log-rank test, and Pearson correlation analysis. Figure S2: the heatmap of gene sets for the top3 GSEA pathway of three hub genes. (A) The heatmap of gene sets for the top3 GSEA pathway of MCM4. (B) The heatmap of gene sets for the top3 GSEA pathway of CENPI. (C) The heatmap of gene sets for the top3 GSEA pathway of KNTC1. Figure S3: the MCM4/6/7 helicase inhibitor heliquinomycin suppresses DU145 cell proliferation by inducing cell cycle arrest in G1 phase. (A) CCK-8 assay evaluates the cell viability of DU145 cells after treatment with heliquinomycin. (B) Cell cycle distribution analysis of DU145 cells after treatment with heliquinomycin using flow cytometry. P values were obtained by Student's t-test. Bars represent the means ± SD of three replicates. ∗P ≤ 0.05, ∗∗P ≤ 0.01, and ∗∗∗P ≤ 0.001. Table S1: the most enriched pathways in top4 modules. Table S2: the list of top sixty-four ranking nodes in the whole PPI network according to the maximal clique centrality (MCC) method. [file 8761112.f1.zip › Supplementary Table 2.pdf]

Supplementary Table 2: The list of top sixty-four ranking nodes in the whole PPI network according to the Maximal Clique Centrality (MCC) method.

Gene	Betweenness	BottleNeck	Closeness	Clustering	Degree	DMNC	EcCentricity	EPC	MCC	MNC	Radiality	Stress
CDK1	11861.6	128	167.4952	0.39581	89	0.76685	0.13316	212.034	9.22E+13	88	11.59125	135740
AURKA	8252.023	13	153.7048	0.5584	69	1.00463	0.11652	212.034	9.22E+13	68	11.43441	93686
CDCA8	799.1509	2	146.2226	0.68651	64	1.1766	0.11652	212.034	9.22E+13	64	11.28054	31076
CDC20	978.1769	2	149.0905	0.58149	71	1.02974	0.11652	212.034	9.22E+13	71	11.24798	14802
BUB1	572.1573	2	147.8905	0.64903	67	1.12856	0.11652	212.034	9.22E+13	67	11.2539	14522
MCM7	208.9875	2	127.4738	0.65909	33	0.91223	0.11652	212.034	9.22E+13	33	11.05564	5942
TPX2	418.0656	1	142.4405	0.69508	61	1.17335	0.11652	212.034	9.22E+13	61	11.174	8314
MCM3	766.5	8	132.4738	0.58583	43	0.88422	0.11652	212.034	9.22E+13	43	11.08523	11490
CDC6	2297.961	3	144.6143	0.56261	58	0.96271	0.11652	212.034	9.22E+13	57	11.26574	31440
BUB1B	710.952	3	148.1405	0.63953	67	1.11204	0.11652	212.034	9.22E+13	67	11.25982	11978
CCNA2	2166.659	3	154.181	0.54572	74	1.00196	0.11652	212.034	9.22E+13	73	11.35156	29540
NEK2	357.6481	2	137.7738	0.74887	52	1.20155	0.11652	212.034	9.22E+13	52	11.14441	4074
CKS2	21.26007	1	128.3405	0.87302	36	1.24351	0.11652	212.034	9.22E+13	36	11.05564	458
CDC25C	542.4978	1	135.881	0.80952	42	1.21258	0.11652	212.034	9.22E+13	42	11.21839	8990
CCNB1	6929.893	1	161.5131	0.49883	78	0.90981	0.11652	212.034	9.22E+13	78	11.54391	122376
SMC4	374.9632	2	130.9738	0.75256	40	1.10952	0.11652	212.034	9.22E+13	40	11.07635	8642
CCNB2	1308.348	1	151.4893	0.60414	70	1.0651	0.11652	212.034	9.22E+13	70	11.3486	40406
BIRC5	783.9766	1	145.7226	0.69176	63	1.17971	0.11652	212.034	9.22E+13	63	11.27758	30646
NCAPG2	55.5892	1	121.6667	0.85714	28	1.12299	0.11652	212.034	9.22E+13	28	10.92839	2232
NCAPG	530.6429	1	146.5738	0.64196	66	1.11099	0.11652	212.034	9.22E+13	66	11.22135	12638
NCAPH	46.62896	1	129.6667	0.80731	43	1.21852	0.11652	212.034	9.22E+13	43	10.98165	856
UBE2C	4865.306	2	149.181	0.66631	62	1.16228	0.11652	212.034	9.22E+13	61	11.35156	48902
CENPE	525.8852	1	140.2405	0.72013	56	1.18307	0.11652	212.034	9.22E+13	56	11.17104	26002
MCM4	956.7218	4	137.3905	0.62039	51	0.98925	0.11652	212.034	9.22E+13	51	11.13553	17624
DLGAP5	76.04056	1	140.8905	0.7633	59	1.27495	0.11652	212.034	9.22E+13	59	11.15329	3008
PTTG1	2111.887	14	141.681	0.8298	51	1.36847	0.11652	212.034	9.22E+13	50	11.27758	28968
RACGAP1	477.6515	3	137.9071	0.80867	49	1.27306	0.11652	212.034	9.22E+13	49	11.16809	24540
KIF20A	477.6409	1	142.7738	0.73005	61	1.23238	0.11652	212.034	9.22E+13	61	11.17992	23460
CENPF	127.5246	1	140.8738	0.76566	57	1.26498	0.11652	212.034	9.22E+13	57	11.17992	4080
TACC3	619.2696	1	124.0738	0.91	25	1.14727	0.11652	212.034	9.22E+13	25	11.06747	8948
CENPA	713.8182	2	139.3738	0.77987	54	1.30736	0.11652	212.034	9.22E+13	53	11.17104	8154
KIF2C	843.6791	3	144.3738	0.68049	63	1.1605	0.11652	212.034	9.22E+13	63	11.20656	33348
NUSAP1	199.4726	1	141.8238	0.74226	59	1.23981	0.11652	212.034	9.22E+13	59	11.17992	5190
FOXM1	1641.029	1	145.7976	0.74563	54	1.21086	0.11652	212.034	9.22E+13	54	11.33972	29280

PBK	75.14467	1	140.4405	0.7901	57	1.30535	0.11652	212.034	9.22E+13	57	11.16217	2506
TOP2A	1431.603	1	149.6393	0.62423	68	1.09052	0.11652	212.034	9.22E+13	68	11.31605	41620
KIF15	544.1295	3	140.9071	0.76094	55	1.24297	0.11652	212.034	9.22E+13	55	11.18584	27036
KIF4A	392.579	1	140.8905	0.75378	58	1.25225	0.11652	212.034	9.22E+13	58	11.15921	22158
KIF18B	0.79968	1	122.6988	0.9871	31	1.33813	0.11652	212.034	9.22E+13	31	10.93431	38
ASPM	227.8415	1	143.2405	0.73107	60	1.22766	0.11652	212.034	9.22E+13	60	11.19768	5398
FANCI	1769.056	3	140.1726	0.71373	51	1.13808	0.11652	212.034	9.22E+13	51	11.24207	29840
CENPM	59.98408	1	133.7071	0.83942	43	1.26699	0.11652	212.034	9.22E+13	43	11.13553	1848
PRC1	200.6621	1	137.0571	0.82431	51	1.31442	0.11652	212.034	9.22E+13	51	11.13257	4892
KIFC1	5.1271	1	126.4571	0.94296	34	1.31808	0.11652	212.034	9.22E+13	34	11.02604	210
KNTC1	25.23062	1	123.5083	0.88046	30	1.18057	0.11652	212.034	9.22E+13	30	10.95206	674
KIF14	6.72459	1	131.2571	0.95488	41	1.41915	0.11652	212.034	9.22E+13	41	11.08523	274
RRM2	335.6652	1	144.6738	0.71356	60	1.19825	0.11652	212.034	9.22E+13	60	11.23319	6966
CENPI	3.42353	1	116.7083	0.95789	20	1.11769	0.11652	212.034	9.22E+13	20	10.884	82
DTL	223.6345	1	139.4071	0.75264	52	1.2076	0.11652	212.034	9.22E+13	52	11.17696	5850
CDKN3	36.35154	1	134.8071	0.88406	46	1.36374	0.11652	212.034	9.22E+13	46	11.1237	828
GTSE1	2.97084	1	126.2905	0.94296	34	1.31808	0.11652	212.034	9.22E+13	34	11.02308	96
MKI67	151.6116	1	139.1238	0.83294	51	1.32817	0.11652	212.034	9.22E+13	51	11.18288	3718
ERCC6L	30.88409	1	121.875	0.84656	28	1.10913	0.11652	212.034	9.22E+13	28	10.92839	706
MELK	205.2432	1	143.1238	0.74576	59	1.24567	0.11652	212.034	9.22E+13	59	11.20656	5518
CDCA3	30.37535	1	131.9238	0.8804	43	1.32883	0.11652	212.034	9.22E+13	43	11.08227	820
TK1	2506.561	5	121.8071	0.8381	21	1.08084	0.11652	212.034	9.22E+13	20	11.03492	29786
XPO1	2105.068	2	125.7714	0.63203	22	0.89661	0.11652	212.034	9.22E+13	20	11.19176	21636
KIAA0101	212.5643	1	135.9905	0.81869	47	1.27168	0.11652	212.034	9.22E+13	47	11.14737	4408
ASF1B	11.50083	1	130.4238	0.90553	39	1.32408	0.11652	212.034	9.22E+13	39	11.07931	500
SPAG5	21.31167	1	136.2238	0.8751	50	1.38658	0.11652	212.034	9.22E+13	50	11.1237	960
RAD54L	89.13782	1	126.0488	0.83601	34	1.16858	0.11652	212.034	9.22E+13	34	10.99053	3076
TROAP	18.1076	1	128.1861	0.91465	38	1.32611	0.10357	212.034	9.22E+13	38	11.02012	780
PPP2R5B	1931.597	1	125.4976	0.73684	20	0.85976	0.11652	212.034	9.22E+13	20	11.18288	39192
FAM64A	2.93987	1	127.2905	0.97479	35	1.37566	0.11652	212.034	9.22E+13	35	11.0438	190
